# *MIR17HG* polymorphism (rs7318578) is associated with liver cancer risk in the Chinese Han population

**DOI:** 10.1042/BSR20193312

**Published:** 2020-08-28

**Authors:** Xu Chao, Xuesong Feng, Hailong Shi, Yuewen Wang, Lanlan Wang, Haiyu Shen, Qing Zha, Yanni Chen, Chao Jiang

**Affiliations:** 1 The Second Affiliated Hospital, Shaanxi University of Chinese Medicine, Xianyang, Shaanxi 712000, China; 2 The College of Basic medicine, Shaanxi University of Chinese Medicine, Xianyang, Shaanxi 712046, China; 3 The Second Affiliated Hospital of Xi'an Medical University, Xi’an, Shaanxi 710038, China; 4 Longhua Hospital, Shanghai University of traditional Chinese Medicine, Shanghai 200032, China

**Keywords:** case-control study, liver cancer, MIR17HG, single nucleotide polymorphisms, susceptibility

## Abstract

Numerous evidence has revealed that single-nucleotide polymorphisms (SNPs) are associated with liver cancer risk. To assess whether the *MIR17HG* polymorphisms are associated with the liver cancer risk in the Chinese Han population, we performed a case–control (432 liver cancer patients and 430 healthy controls) study. Genotyping of four variants of *MIR17HG* was performed with the Agena MassARRAY platform. We used χ^2^ test to compare the distribution of SNPs allele and genotypes frequencies of cases and controls. Odds ratios (ORs) and 95% confidence intervals (CIs) were calculated by logistic regression analysis to evaluate the association under genetic models. The results indicated that the rs7318578 was significantly associated with increased the risk of liver cancer in the allele (OR = 1.45, 95% CI: 1.18–1.77, *P*=3.04E-04), recessive (OR = 3.69, 95% CI: 2.45–5.56, *P*=4.52E-10) and additive model (OR = 1.35, 95% CI: 1.13–1.62, *P*=0.001). Moreover, we found that individuals with the genotype CC of rs7318578 presented with an increased risk of liver cancer (OR = 3.03, 95% CI: 1.98–4.65, *P*=3.83E-07); however, the CA genotype of rs7318578 significantly decreased the risk of liver cancer (OR = 0.61, 95% CI: 0.45–0.83, *P*=0.001, compared with those with the AA genotype. Our findings indicated that *MIR17HG* polymorphism (rs7318578) contributes to liver cancer susceptibility to the Chinese Han population. Further studies with larger samples are required to confirm the results, as well as functional studies to determine the role of this SNP in miRNA expression or molecular pathways.

## Introduction

Liver cancer is predicted to be the sixth most commonly diagnosed cancer and the fourth leading cause of cancer death worldwide in 2018, with about 841,000 new cases and 782,000 deaths annually [1]. Liver cancer is also commonly diagnosed and identified as leading causes of cancer death in China, with an estimated about 392,868 newly liver cancer cases and 368,960 death in 2018 [2]. The carcinogenesis of liver cancer is a complex and multistep process regulated by various risk factors. Epidemiological studies indicated that the major environmental risk factors of liver cancer include chronic infections of hepatitis B virus (HBV) and hepatitis C virus, exposure to aflatoxin, alcohol consumption and cigarette smoking, and diabetes [3,4]. Although many individuals are exposed to these risk factors, only a small group of exposed people eventually develop liver cancer, suggesting that host genetic factors may affect liver cancer development. Recently, numerous evidences have revealed that single-nucleotide polymorphisms (SNPs) are associated with liver cancer risk [5–8].

MicroRNAs (miRNAs) are small noncoding single-stranded RNA molecules of about 22 nucleotides, which can regulate target gene expression through complementary binding to their 3′ untranslated region with their seed sequences [9]. MiRNAs have been found to regulate various functions during cancer development, including cancer cell growth, metastasis, cell cycle, apoptosis, invasion, and chemo-resistance [10,11]. The human miRNA 17-92 cluster host gene (MIR17HG) located on chromosome 13q31.3 in the third intron of the c13orf25 (chromosome 13 open reading frame 25) gene, encompasses six miRNAs (miR-17, miR-18a, miR-19a, miR-20a, miR-19b-1 and miR-92a-1) over ∼800 nucleotides. These miRNAs were previously identified to be highly expressed in various types of human cancers, such as lung cancer [12], breast cancer [13], colon cancer [14], pancreatic cancer [15] and gastric cancer [16]. It has demonstrated that miR-92a highly expressed in hepatocellular carcinoma (HCC). In addition, the proliferation of HCC-derived cell lines was enhanced by miR-92a and inhibited by the anti-miR-92a antagomir [17]. A systematic evaluation of candidate oncomiRs and found that up-regulation of miR-18a in HCC was associated with poor patient survival and promoted proliferation in HCC cell lines [18]. However, it remains unclear the mechanism by which miR-17-92 cluster is involved in hepatocellular carcinogenesis.

It has been reported that the *MIR17HG* polymorphisms were associated with the risk of breast cancer [19], colorectal cancer [20,21] and multiple myeloma [22]. However, the association of the SNPs (rs75267932, rs72640334, rs7318578 and rs17735387) in *MIR17HG* with liver cancer susceptibility has not been investigated. Therefore, we performed a case–control (432 liver cancer patients and 430 healthy controls) study to assess whether these four genetic variants are associated with the risk of liver cancer in the Chinese Han population.

## Materials and methods

### Participants

The present study recruited 862 unrelated subjects that visited the Second Affiliated Hospital of Shaanxi University of Chinese Medicine, including 432 patients with liver cancer and 430 healthy control individuals. All the patients with liver cancer were diagnosed by either histopathologic or imaging evidence based on the standards established by the Chinese Society of Liver Cancer (CSLC). The controls are selected from undergoing routine medical examination, which has been excluded those with medical history of surgery, cancer and other diseases. All subjects were unrelated Chinese Han nationality.

### DNA extraction

Genomic DNA was extracted from stored blood using the GoldMag-Mini Whole Blood Genomic DNA Purification Kit (GoldMag. Co. Ltd., Xi’an, China) depending on the manufacturer’s instructions [23,24]. The concentration and purity of extracted DNA determined using a spectrophotometer (NanoDrop 2000; Thermo Fisher Scientific, Waltham, MA, U.S.A.).

### Genotyping

We selected four SNPs (rs75267932, rs72640334, rs7318578 and rs17735387) in *MIR17HG* with a minor allele frequency (MAF) >5% in the global population from the HapMap database. The primers for polymerase chain reaction (PCR) amplification and single base extension of the three SNPs were designed by the Agena Bioscience Assay Design Suite V2.0 software (https://agenacx.com/online-tools/). The four SNPs genotyping were performed using the Agena MassARRAY platform with iPLEX gold chemistry (Agena Bioscience, San Diego, CA, U.S.A.) according to the manufacturer’s instructions. Data were managed and analyzed using the version 4.0 Agena Bioscience TYPER software.

### Statistical analysis

We used the SPSS 20.0 statistical package (SPSS, Chicago, IL) to conduct the basic descriptive statistical analysis of demographic. The chi-squared (χ^2^) test was used to assess the differences in distribution of gender between the case and control groups. The Student’s *t*-test analysis was used to compare the distribution of age between liver cancer patients and controls. The Chi-square analysis was also used to confirm that the genotype distribution of each SNP among the control group was in Hardy–Weinberg equilibrium (HWE). The association for polymorphisms in *MIR17HG* and the liver cancer was assessed under the genetic models (codominant, dominant, recessive and additive) by PLINK software (version 1.07). The genotype and allele frequencies of the four SNPs were compared between liver cancer patients and control subjects with the chi-square test, and the odds ratios (ORs) and 95% confidence intervals (CIs) were estimated using logistic regression analysis. All statistical analyses were two sided and *P*-value of less than 0.05 was considered statistically significant.

## Results

Table 1 shows the statistical analysis of the demographic characteristics of study participants. There were 432 patients with liver cancer (344 males and 88 females) and 430 healthy controls (342 males and 88 females) in the present study. The average ages of cases and controls were 55.09 years old and 55.22 years old, respectively. No significant differences existed between the case group and the control group in regard to gender, smoking, and drinking (*P*>0.05). Similarly, no significant differences were found in terms of mean age (*P*= 0.861) and BMI (*P*=0.571) between these two groups.

**Table 1:** Characteristics of liver cancer patients and controls in the present study

Characteristics	Cases (%)	Controls (%)	*P*
Total	432	430	
Gender			
Male	344 (79.6)	342 (79.5)	0.846
Female	88 (20.4)	88 (20.5)	
Age (year)			
Mean ± SD	55.09 ± 11.59	55.22 ± 10.73	0.861
BMI			
Mean ± SD	24.52 ± 4.52	24.36 ± 4.29	0.571
Smoking			
Yes	183 (42.4)	173 (40.2)	0.556
No	249 (57.6)	257 (59.8)	
Drinking			
Yes	168 (38.9)	162 (37.7)	0.714
No	264 (61.1)	268 (62.3)	
Tumor history			
Yes	50 (11.6)	18 (4.2)	<0.001
No	382 (88.4)	412 (95.8)	
Tumor stage			
I-II	274 (63.0)		
III-IV	161 (37.0)		

*P*<0.05 indicates statistical significance.

Four selected SNPs in *MIR17HG* were successfully genotyped (call rate >95%). Allele distributions and associations of the *MIR17HG* gene polymorphisms with liver cancer risk are shown in Table 2. The genotypes of rs75267932, rs72640334, rs7318578 and rs17735387 were in agreement with the HWE in control group (*P*>0.05). In the overall analysis, we found that the allele C of rs7318578 with the higher distribution frequency in the controls than cases (0.377 vs. 0.294, *P*=3.04E-04). The SNP rs7318578 was associated with higher risk of liver cancer with an OR 1.45 (95% CI: 1.18–1.77). The association remained significant even after Bonferroni correction, the significant level is 0.05/4 = 0.0125. However, no significant association was observed between the three SNPs in *MIR17HG* (rs75267932, rs72640334 and rs17735387) and liver cancer risk.

**Table 2:** Allele frequencies distribution and association with liver cancer risk

SNP–ID	Chr, Position	Role	Allele A/B	MAF		HWE–*P*	OR (95%CI)	*P*
				Case	Control			
rs75267932	13, 91351812	Intron	G/A	0.116	0.111	0.461	1.04 (0.78–1.41)	0.775
rs72640334	13, 91352674	Intron	A/C	0.115	0.088	0.560	1.34 (0.98–1.83)	0.069
rs7318578	13, 91353215	Intron	C/A	0.377	0.294	0.727	1.45 (1.18–1.77)	3.04E-04
rs17735387	13, 91353800	Intron	A/G	0.201	0.201	0.652	1.00 (0.79–1.27)	0.971

Abbreviations: CI, confidence interval; Chr, chromosome; HWE, Hardy–Weinberg equilibrium; MAF, minor allele frequency; OR: odds ratio; SNP, single-nucleotide polymorphism.

*P*<0.05 indicates statistical significance.

*P*<(0.05/4 = 0.0125) indicates statistical significance with Bonferroni correction.

To explore the association between the genotype distributions and associations of the four SNPs in *MIR17HG* with liver cancer risk, we performed four genetic models (codominant, dominant, recessive and additive) analysis before and after adjusted with age and gender, as shown in Table 3. Individuals with the homozygous genotype CC of rs7318578 presented with an increased risk of liver cancer, compared with those with the AA genotype (OR = 3.01, 95% CI: 1.97–4.62, *P*=4.09E-07; adjusted OR = 3.03, 95% CI: 1.98–4.65, *P*=3.83E-07). The rs7318578 was also found to be associated with an increased with liver cancer risk in the recessive model (OR = 3.67, 95% CI: 2.44–5.53, *P*=4.70E-10; adjusted OR = 3.69, 95% CI: 2.45–5.56, *P*=4.52E-10) and the additive model (OR = 1.35, 95% CI: 1.13–1.63, *P*=0.001; adjusted OR = 1.35, 95% CI: 1.13–1.62, *P*=0.001). However, the results showed that CA genotype of rs7318578 was associated with a decreased risk of liver cancer, compared with those with the AA genotype (OR = 0.61, 95% CI: 0.45–0.83, *P*=0.002; adjusted OR = 0.61, 95% CI: 0.45–0.83, *P*=0.001). The association also remained significant even after Bonferroni correction (0.05/4 = 0.0125). However, no any significant association was found between the SNPs (rs75267932, rs72640334 and rs17735387) in *MIR17HG* and risk of liver cancer.

**Table 3:** Genetics models analyses of association *MIR17HG* polymorphisms with liver cancer risk

SNP-ID	Model	Genotype	Case (%)	Control (%)	OR (95% CI)	*P*	adjust OR (95% CI)	*P*
rs75267932	Codominant	AA	340 (78.7)	342 (79.4)	1		1	
		GA	84 (19.4)	82 (19.0)	1.03 (0.73–1.45)	0.863	1.03 (0.74–1.45)	0.850
		GG	8 (1.9)	7 (1.6)	1.15 (0.41–3.21)	0.790	1.15 (0.41–3.21)	0.788
	Dominant	AA	340 (78.7)	342 (79.4)	1		1	
		GG-GA	92 (21.3)	89 (20.6)	1.04 (0.75–1.44)	0.816	1.04 (0.75–1.45)	0.803
	Recessive	GA-AA	424 (98.1)	424 (98.4)	1.14 (0.41–3.18)	0.798	1.14 (0.41–3.18)	0.797
		GG	8 (1.9)	7 (1.6)				
	Additive				1.04 (0.78–1.4)	0.779	1.05 (0.78–1.40)	0.768
rs72640334	Codominant	CC	336 (77.8)	357 (82.8)	1		1	
		AC	93 (21.5)	72 (16.7)	1.37 (0.98–1.93)	0.070	1.37 (0.97–1.93)	0.070
		AA	3 (0.7)	2 (0.5)	1.59 (0.26–9.60)	0.611	1.59 (0.26–9.60)	0.613
	Dominant	CC	336 (77.8)	357 (82.8)	1		1	
		AA-AC	96 (22.2)	74 (17.2)	1.38 (0.98–1.93)	0.063	1.38 (0.98–1.93)	0.064
	Recessive	AC-CC	429 (99.3)	429 (99.5)	1		1	
		AA	3 (0.7)	2 (0.5)	1.50 (0.25–9.02)	0.658	1.49 (0.25–9.00)	0.662
	Additive				1.36 (0.98–1.88)	0.063	1.36 (0.98–1.88)	0.064
rs7318578	Codominant	AA	212 (49.3)	211 (49.3)	1		1	
		CA	112 (26.0)	182 (42.5)	0.61 (0.45–0.83)	0.002	0.61 (0.45–0.83)	0.001
		CC	106 (24.7)	35 (8.2)	3.01 (1.97–4.62)	4.09E–07	3.03 (1.98–4.65)	3.83E–07
	Dominant	AA	212 (49.3)	211 (49.3)	1		1	
		CC-CA	218 (50.7)	217 (50.7)	1.00 (0.77–1.31)	0.999	1.00 (0.76–1.31)	0.996
	Recessive	CA-AA	324 (75.3)	393 (91.8)	1		1	
		CC	106 (24.7)	35 (8.2)	3.67 (2.44–5.53)	4.70E–10	3.69 (2.45–5.56)	4.52E–10
	Additive				1.35 (1.13–1.63)	0.001	1.35 (1.13–1.62)	0.001
rs17735387	Codominant	GG	276 (63.9)	277 (64.3)	1		1	
		AG	138 (31.9)	135 (31.3)	1.03 (0.77–1.37)	0.863	1.03 (0.77–1.37)	0.867
		AA	18 (4.2)	19 (4.4)	0.95 (0.49–1.85)	0.882	0.95 (0.49–1.85)	0.880
	Dominant	GG	276 (63.9)	277 (64.3)	1		1	
		AA-AG	156 (36.1)	154 (35.7)	1.02 (0.77–1.34)	0.907	1.02 (0.77–1.34)	0.912
	Recessive	AG-GG	414 (95.8)	412 (95.6)	1		1	
		AA	18 (4.2)	19 (4.4)	0.94 (0.49–1.82)	0.861	0.94 (0.49–1.82)	0.859
	Additive				1.00 (0.80–1.27)	0.972	1.00 (0.79–1.27)	0.976

Abbreviations: CI, confidence interval; OR, odds ratio; SNP, single-nucleotide polymorphism.

Adjust OR (95% CI) were calculated by logistic regression analysis with adjustments for age and gender.

*P*<0.05 indicates statistical significance.

*P*<(0.05/4 = 0.0125) indicates statistical significance with Bonferroni correction.

We further divided the data into subgroups based on age, gender, smoking, drinking and BMI (Table 4). When stratifying by age, we found that the genotype CC of rs7318578 was significantly associated with an increased risk of liver cancer, compared with the AA genotype and CA-AA genotype in age >55 years old (OR = 3.37, 95% CI: 1.74–6.51, *P*<0.0001; OR = 3.91, 95% CI: 2.07–7.38, *P*<0.0001); age ≤55 years old (OR = 2.73, 95% CI: 1.55–4.83, *P*=0.001; OR = 3.43, 95% CI: 1.99–5.92, *P*<0.0001); males (OR = 2.77, 95% CI: 1.72–4.46, *P*<0.0001; OR = 3.44, 95% CI: 2.18–5.42, *P*<0.0001); females (OR = 4.30, 95% CI: 1.60–11.60, *P*=0.004; OR = 4.93, 95% CI: 1.89–12.84, *P*=0.001); no smoking (OR = 3.46, 95% CI: 1.90–6.30, *P*<0.0001; OR = 4.13, 95% CI: 2.32–7.33, *P*<0.0001); smoking (OR = 2.72, 95% CI: 1.46–5.07, *P*<0.0001; OR = 3.43, 95% CI: 1.89–6.23, *P*<0.0001); no drinking (OR = 2.96, 95% CI: 1.70–5.17, *P*<0.0001; OR = 3.72, 95% CI: 2.17–6.37, *P*<0.0001); drinking (OR = 3.30, 95% CI: 1.67–6.52, *P*<0.0001; OR = 3.87, 95% CI: 2.03–7.38, *P*<0.0001); BMI > 24 (OR = 3.32, 95% CI: 1.76–6.25, *P*<0.0001; OR = 4.46, 95% CI: 2.43–8.18, *P*<0.0001); BMI ≤ 24 (OR = 2.88, 95% CI: 1.60–5.21, *P*<0.0001; OR = 3.24, 95% CI: 1.84–5.72, *P*<0.0001).

**Table 4:** Association of rs7318578 with liver cancer risk

Model	Genotype	Case (%)	Control (%)	OR (95% CI)	*P*	case	control	OR (95% CI)	*P*
Age		>55				≤55			
Codominant	AA	101 (48.6)	94 (50.8)	1		111 (50)	117 (48.1)	1	
	CA	57 (27.4)	77 (41.6)	0.68 (0.44–1.07)	0.094	55 (24.8)	105 (43.2)	0.56 (0.37–0.86)	0.007
	CC	50 (24)	14 (7.6)	3.37 (1.74–6.51)	<0.0001	56 (25.2)	21 (8.6)	2.73 (1.55–4.83)	0.001
Dominant	AA	101 (48.6)	94 (50.8)	1		111 (50)	117 (48.1)	1	
	CC-CA	107 (51.4)	91 (49.2)	1.09 (0.74–1.63)	0.658	111 (50)	126 (51.9)	0.93 (0.64–1.34)	0.698
Recessive	CA-AA	158 (76)	171 (92.4)	1		166 (74.8)	222 (91.4)	1	
	CC	50 (24)	14 (7.6)	3.91 (2.07–7.38)	<0.0001	56 (25.2)	21 (8.6)	3.43 (1.99–5.92)	<0.0001
Additive	—	—	—	1.41 (1.08–1.86)	0.013	—	—	1.29 (1.01–1.66)	0.043
Gender		Male				Female			
Codominant	AA	170 (49.6)	163 (48.1)	1		42 (48.3)	47 (53.4)	1	
	CA	90 (26.2)	147 (43.4)	0.59 (0.42–0.82)	0.002	22 (25.3)	35 (39.8)	0.70 (0.36–1.38)	0.306
	CC	83 (24.2)	29 (8.6)	2.77 (1.72–4.46)	<0.0001	23 (26.4)	6 (6.8)	4.30 (1.6–11.6)	0.004
Dominant	AA	170 (49.6)	163 (48.1)	1		42 (48.3)	47 (53.4)	1	
	CC-CA	173 (50.4)	176 (51.9)	0.94 (0.70–1.27)	0.698	45 (51.7)	41 (46.6)	1.23 (0.68–2.22)	0.497
Recessive	CA-AA	260 (75.8)	310 (91.4)	1		64 (73.6)	82 (93.2)	1	
	CC	83 (24.2)	29 (8.6)	3.44 (2.18–5.42)	<0.0001	23 (26.4)	6 (6.8)	4.93 (1.89–12.84)	0.001
Additive	—	—	—	1.30 (1.06–1.59)	0.013	—	—	1.57 (1.05–2.37)	0.030
Smoking		No				Yes			
Codominant	A/A	118 (48)	124 (48.6)	1	<0.0001	93 (50.8)	86 (50)	1	<0.0001
	C/A	72 (29.3)	114 (44.7)	0.66 (0.45–0.98)		40 (21.9)	69 (40.1)	0.54 (0.33–0.87)	
	C/C	56 (22.8)	17 (6.7)	3.46 (1.90–6.30)		50 (27.3)	17 (9.9)	2.72 (1.46–5.07)	
Dominant	A/A	118 (48)	124 (48.6)	1	0.880	93 (50.8)	86 (50)	1	0.880
	C/A-C/C	128 (52)	131 (51.4)	1.03 (0.72–1.46)		90 (49.2)	86 (50)	0.97 (0.64–1.47)	
Recessive	A/A-C/A	190 (77.2)	238 (93.3)	1	<0.0001	133 (72.7)	155 (90.1)	1	<0.0001
	C/C	56 (22.8)	17 (6.7)	4.13 (2.32–7.33)		50 (27.3)	17 (9.9)	3.43 (1.89–6.23)	
Additive	—	—	—	1.39 (1.08–1.78)	0.009	—	—	1.33 (1.01–1.74)	0.042
Drinking		No				Yes			
Codominant	A/A	139 (53)	135 (50.9)	1	<0.0001	73 (43.5)	75 (46.3)	1	<0.0001
	C/A	62 (23.7)	110 (41.5)	0.55 (0.37–0.81)		50 (29.8)	73 (45.1)	0.70 (0.43–1.14)	
	C/C	61 (23.3)	20 (7.5)	2.96 (1.70–5.17)		45 (26.8)	14 (8.6)	3.30 (1.67–6.52)	
Dominant	A/A	139 (53)	135 (50.9)	1	0.630	73 (43.5)	75 (46.3)	1	0.600
	C/A-C/C	123 (47)	130 (49.1)	0.92 (0.65–1.29)		95 (56.5)	87 (53.7)	1.12 (0.73–1.73)	
Recessive	A/A-C/A	201 (76.7)	245 (92.5)	1	<0.0001	123 (73.2)	148 (91.4)	1	<0.0001
	C/C	61 (23.3)	20 (7.5)	3.72 (2.17–6.37)		45 (26.8)	14 (8.6)	3.87 (2.03–7.38)	
Additive	—	—	—	1.29 (1.02–1.63)	0.033	—	—	1.47 (1.09–1.97)	0.010
BMI		>24				≤ 24			
Codominant	A/A	108 (49.1)	96 (45.5)	1	<0.0001	104 (49.5)	114 (52.8)	1	<0.0001
	C/A	56 (25.4)	100 (47.4)	0.50 (0.32–0.76)		56 (26.7)	83 (38.4)	0.74 (0.48–1.14)	
	C/C	56 (25.4)	15 (7.1)	3.32 (1.76–6.25)		50 (23.8)	19 (8.8)	2.88 (1.60–5.21)	
Dominant	A/A	108 (49.1)	96 (45.5)	1	0.460	104 (49.5)	114 (52.8)	1	0.500
	C/A-C/C	112 (50.9)	115 (54.5)	0.87 (0.59–1.26)		106 (50.5)	102 (47.2)	1.14 (0.78–1.67)	
Recessive	A/A-C/A	164 (74.5)	196 (92.9)	1	<0.0001	160 (76.2)	197 (91.2)	1	<0.0001
	C/C	56 (25.4)	15 (7.1)	4.46 (2.43–8.18)		50 (23.8)	19 (8.8)	3.24 (1.84–5.72)	
Additive	—	—	—	1.32 (1.01–1.70)	0.037	—	—	1.40 (1.08–1.81)	0.011

Abbreviations: CI, confidence interval; OR, odds ratio; SNP, single-nucleotide polymorphism.

OR (95% CI) were calculated by logistic regression analysis with adjustments for age and gender.

*P*<0.05 indicates statistical significance.

*P*<(0.05/4 = 0.0125) indicates statistical significance with Bonferroni correction.

In the additive model, rs7318578 was found to be associated with significantly increased risk of liver cancer in the age >55 years old (OR = 1.41, 95% CI: 1.08–1.86, *P*=0.013), age ≤55 years old (OR = 1.29, 95% CI: 1.01–1.66, *P*=0.043); males (OR = 1.30, 95% CI: 1.06–1.59, *P*=0.013), females (OR = 1.57, 95% CI: 1.05–2.37, *P*=0.030); no smoking (OR = 1.39, 95% CI: 1.08–1.78, *P*=0.009), smoking (OR = 1.33, 95% CI: 1.01–1.74, *P*=0.042); no drinking (OR = 1.29, 95% CI: 1.02–1.63, *P*=0.033), drinking (OR = 1.47, 95% CI: 1.09–1.97, *P*=0.010); BMI > 24 (OR = 1.32, 95% CI: 1.01–1.70, *P*=0.037), BMI ≤ 24 (OR = 1.40, 95% CI: 1.08–1.81, *P*=0.011) (Table 4).

However, the CA genotype of rs7318578 significantly decreased the risk of liver cancer in age ≤55 years old (OR = 0.56, 95% CI: 0.37–0.86, *P*=0.007); males (OR = 0.59, 95% CI: 0.42–0.82, *P*=0.002); no smoking (OR = 0.66, 95% CI: 0.45–0.98, *P*<0.0001), smoking (OR = 0.54, 95% CI: 0.33–0.87, *P*<0.0001); no drinking (OR = 0.55, 95% CI: 0.37–0.81, *P*<0.0001); BMI > 24 (OR = 0.50, 95% CI: 0.32–0.76, *P*<0.0001), compared with the AA genotype (Table 4).

## Discussion

In the present study, we investigated the association between *MIR17HG* polymorphisms and liver cancer risk in the Chinese Han population. Overall, stratification analysis found that the rs7318578 was significantly associated with increased the risk of liver cancer in allele, recessive and additive models. Overall, stratification analysis results indicated that individuals with the homozygous genotype CC of rs7318578 presented with an increased risk of liver cancer; however, the CA genotype of rs7318578 significantly decreased the risk of liver cancer in overall, male and age ≤55 years old, compared with those with the AA genotype.

This *MIR17HG* gene is the host gene for the MIR17-92 cluster, a group of at least six microRNAs (miRNAs) that may be involved in cell survival, proliferation, differentiation and angiogenesis [25]. The miR-17-92 gene cluster, also known as C13orf25, is closely related to tumorigenesis by inhibiting the expression of cell cycle regulatory genes and tumor suppressor genes. The E2F, p53, STAT3 and c-Myc bind to the promoter region of the miR-17-92 gene cluster and regulate downstream target genes, thereby affecting biological processes such as cell proliferation, invasion, migration and apoptosis, playing an important role in tumorigenesis [26–28]. The oncogenic effect of the miR-17-92 cluster is enhanced by cooperation between its members in targeting tumor-suppressive proteins and pathways such as PTEN and TGFβ signaling [29]. MiR17-92 cluster is an oncogenic miRNA cluster that is implicated in several cancers. The miR-17-92 cluster has been reported to be highly expressed in human hepatocellular carcinoma (HCC) tissues compared with the non-tumorous liver tissues by reverse transcription-polymerase chain reaction (RT-PCR) and *in situ* hybridization analyses. Moreover, forced overexpression of the miR-17-92 cluster in cultured human hepatocellular cancer cells enhanced tumor cell proliferation, colony formation and invasiveness *in vitro*, whereas inhibition of the miR-17-92 cluster reduced tumor cell growth [30]. A recent study shows that the histone deacetylase inhibitors (HDACi) SAHA epigenetically upregulates MICA expression through regulating the expression of miR-17-92 cluster and MCM7 in hepatoma. Thus, enhancing the sensitivity of HCC to natural killer cell-mediated lysis [31]. These findings suggest that miR-17-92 cluster plays a pivotal role in the development of liver cancer. However, its role in hepatocarcinogenesis has not been clearly established.

It has been reported the rs7336610 and AC haplotype of rs4824505/rs7336610 are associated with risk of breast cancer [19]. Polymorphisms (rs7336610 and rs1428), haplotype AC (rs4284505-rs1428) and CA (rs7336610-rs4284505) of *MIR17HG* were correlated with increased multiple myeloma risk, whereas haplotype GC (rs4284505-rs1428) significantly elevated multiple myeloma risk [22]. Meanwhile, Kaplan–Meier curve analysis demonstrated that the CC genotype of rs7336610 and the AA genotype carriers of rs4284505 had higher 5-year survival. Previous study reported that two functional polymorphisms (rs9588884 and rs982873) in the promoter region of miR-17-92 cluster are associated with a decreased risk of colorectal cancer [20]. Recently, Chen et al. [21] indicated that the two SNPs (rs7336610 and rs1428) of *MIR17HG* were associated with increased colorectal cancer risk, and the two SNPs (rs7318578 and rs17735387) of *MIR17HG* were associated with decreased colorectal cancer risk in the Chinese Han population. In the present study, we investigate the association between of polymorphisms of *MIR17HG* and liver cancer risk in the Chinese Han population. The results indicated that the genotype CC of rs7318578 was associated with an increased risk of liver cancer; however, the CA genotype of rs7318578 significantly reduced the risk of liver cancer, compared with those with the AA genotype. In the genomes project in Han Chinese, the frequency of allele C of rs7318578 is 0.277; the frequencies of genotype AA, AC and CC are 0.515, 0.417 and 0.068, respectively. In the present study control group, the frequency of allele C of rs7318578 is 0.294; the frequencies of genotype AA, AC and CC are 0.493, 0.425 and 0.082, respectively. There was no significant difference in the frequency distribution of alleles (*P*=0.616) and genotypes (*P*=0.865) of rs7318578 between this study control group and genomes project CHB. Therefore, the further research is needed to verify the results.

To the best of our knowledge, the present study is the first to assess the association between polymorphisms in the *MIR17HG* gene and liver cancer risk in the Chinese Han population. The limitations of our work should be mentioned. First, the sample size of the present study is relatively small. Second, limited the gene–environment interaction analysis. Third, additional SNPs in *MIR17HG* may be associated with liver cancer but were not assessed for their potential associations. Finally, the present study did not elucidate the role of this SNP in miRNA expression or the specific mechanism of the *MIR17HG* polymorphisms affecting in the development of liver cancer.

## Conclusions

In conclusion, our study provides evidence that polymorphism (rs7318578) in *MIR17HG* was associated with susceptibility to liver cancer in the Chinese Han population. To the best of our knowledge, this is the first time study investigating SNPs in the *MIR17HG* gene in liver cancer. Therefore, our findings are required to confirm in further with larger populations and/or different ethnicities, as well as functional studies to determine the role of this SNP in miRNA expression or molecular pathways affecting in the development of liver cancer.

## Data Availability

The data used to support the findings of this study are available from the corresponding author upon request.

## Abbreviations

CI: confidence interval
HCC: human hepatocellular carcinoma
HDACi: histone deacetylase inhibitors
OR: odds ratio
RT-PCR: reverse transcription-polymerase chain reaction
SNP: single-nucleotide polymorphism

## References

[1] Bray F., Ferlay J., Soerjomataram I., Siegel R.L., Torre L.A. and Jemal A. (2018) Global cancer statistics 2018: GLOBOCAN estimates of incidence and mortality worldwide for 36 cancers in 185 countries. CA Cancer J. Clin. 68, 394–424 10.3322/caac.21492 30207593

[2] Feng R.M., Zong Y.N., Cao S.M. and Xu R.H. (2019) Current cancer situation in China: good or bad news from the 2018 Global Cancer Statistics? Cancer Commun. 39, 22 10.1186/s40880-019-0368-6 PMC6487510 31030667

[3] Gomaa A.I., Khan S.A., Toledano M.B., Waked I. and Taylor-Robinson S.D. (2008) Hepatocellular carcinoma: epidemiology, risk factors and pathogenesis. World J. Gastroenterol. 14, 4300–4308 10.3748/wjg.14.4300 18666317 PMC2731180

[4] Chuang S.C., La Vecchia C. and Boffetta P. (2009) Liver cancer: descriptive epidemiology and risk factors other than HBV and HCV infection. Cancer Lett. 286, 9–14 10.1016/j.canlet.2008.10.040 19091458

[5] Zhou J., Lv R., Song X. et al. (2012) Association between two genetic variants in miRNA and primary liver cancer risk in the Chinese population. DNA Cell Biol. 31, 524–530 10.1089/dna.2011.1340 21861697 PMC3322400

[6] Li H.G., Liu F.F., Zhu H.Q. et al. (2015) Association of PTEN gene polymorphisms with liver cancer risk. Int. J. Clin. Exp. Pathol. 8, 15198–15203 26823866 PMC4713652

[7] Wen J., Song C., Jiang D. et al. (2015) Hepatitis B virus genotype, mutations, human leukocyte antigen polymorphisms and their interactions in hepatocellular carcinoma: a multi-centre case-control study. Sci. Rep. 5, 16489 10.1038/srep16489 26568165 PMC4644975

[8] Chen Z., Sun Y., Xu Z. et al. (2017) ACYP2 polymorphisms are associated with the risk of liver cancer in a Han Chinese population. Oncotarget 8, 67723–67731 10.18632/oncotarget.18574 28978066 PMC5620206

[9] Bartels C.L. and Tsongalis G.J. (2009) MicroRNAs: novel biomarkers for human cancer. Clin. Chem. 55, 623–631 10.1373/clinchem.2008.112805 19246618

[10] Jansson M.D. and Lund A.H. (2012) MicroRNA and cancer. Mol. Oncol. 6, 590–610 10.1016/j.molonc.2012.09.006 23102669 PMC5528350

[11] Hayes J., Peruzzi P.P. and Lawler S. (2014) MicroRNAs in cancer: biomarkers, functions and therapy. Trends Mol. Med. 20, 460–469 10.1016/j.molmed.2014.06.005 25027972

[12] Zhang X., Li Y., Qi P. and Ma Z. (2018) Biology of MiR-17-92 Cluster and Its Progress in Lung Cancer. Int. J. Med. Sci. 15, 1443–1448 10.7150/ijms.27341 30443163 PMC6216058

[13] Li H., Bian C., Liao L., Li J. and Zhao R.C. (2011) miR-17-5p promotes human breast cancer cell migration and invasion through suppression of HBP1. Breast Cancer Res. Treat. 126, 565–575 10.1007/s10549-010-0954-4 20505989

[14] Liu G.H., Zhou Z.G., Chen R. et al. (2013) Serum miR-21 and miR-92a as biomarkers in the diagnosis and prognosis of colorectal cancer. Tumour Biol.: J. Int. Soc. Oncodevelopment. Biol. Med. 34, 2175–2181 10.1007/s13277-013-0753-8 23625654

[15] Chen Y., Tian L., Wan S. et al. (2016) MicroRNA-17-92 cluster regulates pancreatic beta-cell proliferation and adaptation. Mol. Cell. Endocrinol. 437, 213–223 10.1016/j.mce.2016.08.037 27568466

[16] Bahari F., Emadi-Baygi M. and Nikpour P. (2015) miR-17-92 host gene, uderexpressed in gastric cancer and its expression was negatively correlated with the metastasis. Indian J. Cancer 52, 22–25 26837962 10.4103/0019-509X.175605

[17] Shigoka M., Tsuchida A., Matsudo T. et al. (2010) Deregulation of miR-92a expression is implicated in hepatocellular carcinoma development. Pathol. Int. 60, 351–357 10.1111/j.1440-1827.2010.02526.x 20518884

[18] Sanchez-Mejias A., Kwon J., Chew X.H. et al. (2019) A novel SOCS5/miR-18/miR-25 axis promotes tumorigenesis in liver cancer. Int. J. Cancer 144, 311–321 10.1002/ijc.31857 30191950

[19] Chacon-Cortes D., Smith R.A., Lea R.A., Youl P.H. and Griffiths L.R. (2015) Association of microRNA 17-92 cluster host gene (MIR17HG) polymorphisms with breast cancer. Tumour Biol.: J. Int. Soc. Oncodevelopment. Biol. Med. 36, 5369–5376 10.1007/s13277-015-3200-1 25680407

[20] Sun R., Liang Y., Yuan F. et al. (2017) Functional polymorphisms in the promoter region of miR-17-92 cluster are associated with a decreased risk of colorectal cancer. Oncotarget 8, 82531–82540 10.18632/oncotarget.19753 29137282 PMC5669908

[21] Chen P., Bai Y., Li Y. et al. (2019) Association between polymorphisms of MIR17HG and risk of colorectal cancer in the Chinese Han population. Mol. Genet. Genom. Med. e667 10.1002/mgg3.667 PMC6565593 30941921

[22] Wu H., Huang T., Ye Z., Fu X., Hu K. and Yang X. (2019) Correlation of MicroRNA 17-92 Cluster Host Gene (MIR17HG) Polymorphisms With Susceptibility and Prognosis for Multiple Myeloma. Clin. Lymphoma Myeloma Leuk. 19, 359–366 10.1016/j.clml.2019.03.018 31029648

[23] Huang C.Y., Xun X.J., Wang A.J. et al. (2015) CHRNA5 polymorphisms and risk of lung cancer in Chinese Han smokers. Am. J. Cancer Res. 5, 3241–3248 26693074 PMC4656745

[24] Rong H., He X., Zhu L. et al. (2017) Association between regulator of telomere elongation helicase1 (RTEL1) gene and HAPE risk: A case-control study. Medicine (Baltimore) 96, e8222 10.1097/MD.0000000000008222 28953687 PMC5626330

[25] Mogilyansky E. and Rigoutsos I. (2013) The miR-17/92 cluster: a comprehensive update on its genomics, genetics, functions and increasingly important and numerous roles in health and disease. Cell Death Differ. 20, 1603–1614 10.1038/cdd.2013.125 24212931 PMC3824591

[26] Aguda B.D., Kim Y., Piper-Hunter M.G., Friedman A. and Marsh C.B. (2008) MicroRNA regulation of a cancer network: consequences of the feedback loops involving miR-17-92, E2F, and Myc. Proc. Natl. Acad. Sci. U.S.A. 105, 19678–19683 10.1073/pnas.0811166106 19066217 PMC2598727

[27] Nittner D., Lambertz I., Clermont F. et al. (2012) Synthetic lethality between Rb, p53 and Dicer or miR-17-92 in retinal progenitors suppresses retinoblastoma formation. Nat. Cell Biol. 14, 958–965 10.1038/ncb2556 22864477

[28] Jo D.H., Kim J.H., Cho C.S. et al. (2014) STAT3 inhibition suppresses proliferation of retinoblastoma through down-regulation of positive feedback loop of STAT3/miR-17-92 clusters. Oncotarget 5, 11513–11525 10.18632/oncotarget.2546 25359779 PMC4294389

[29] Fuziwara C.S. and Kimura E.T. (2015) Insights into Regulation of the miR-17-92 Cluster of miRNAs in Cancer. Front. Med. 2, 64 10.3389/fmed.2015.00064 PMC4561802 26442266

[30] Zhu H., Han C. and Wu T. (2015) MiR-17-92 cluster promotes hepatocarcinogenesis. Carcinogenesis 36, 1213–1222 10.1093/carcin/bgv112 26233958 PMC4794620

[31] Connolly E., Melegari M., Landgraf P. et al. (2008) Elevated expression of the miR-17-92 polycistron and miR-21 in hepadnavirus-associated hepatocellular carcinoma contributes to the malignant phenotype. Am. J. Pathol. 173, 856–864 10.2353/ajpath.2008.080096 18688024 PMC2527078

